# Long-term maintenance of functional primary human hepatocytes in 3D gelatin matrices produced by solution blow spinning

**DOI:** 10.1038/s41598-021-99659-1

**Published:** 2021-10-11

**Authors:** Mariliis Klaas, Kaidi Möll, Kristina Mäemets-Allas, Mart Loog, Martin Järvekülg, Viljar Jaks

**Affiliations:** 1 Institute of Molecular and Cell Biology, University of Tartu, Riia 23b, 51010 Tartu, Estonia; 2 Institute of Technology, University of Tartu, Nooruse 1, 50411 Tartu, Estonia; 3 Laboratory of Physics of Nanostructures, Institute of Physics, University of Tartu, W. Ostwaldi 1, 50411 Tartu, Estonia; 4 Dermatology Clinic, Tartu University Hospital, Raja 31, 50417 Tartu, Estonia

**Keywords:** Biomaterials, Hepatocytes, Biomaterials - cells

## Abstract

Solution blow spinning (SBS) has recently emerged as a novel method that can produce nano- and microfiber structures suitable for tissue engineering. Gelatin is an excellent precursor for SBS as it is derived mainly from collagens that are abundant in natural extracellular matrices. Here we report, for the first time the successful generation of 3D thermally crosslinked preforms by using SBS from porcine gelatin. These SBS mats were shown to have three-dimensional fibrous porous structure similar to that of mammalian tissue extracellular matrix. In pharma industry, there is an urgent need for adequate 3D liver tissue models that could be used in high throughput setting for drug screening and to assess drug induced liver injury. We used SBS mats as culturing substrates for human hepatocytes to create an array of 3D human liver tissue equivalents in 96-well format. The SBS mats were highly cytocompatible, facilitated the induction of hepatocyte specific CYP gene expression in response to common medications, and supported the maintenance of hepatocyte differentiation and polarization status in long term cultures for more than 3 weeks. Together, our results show that SBS-generated gelatin scaffolds are a simple and efficient platform for use in vitro for drug testing applications.

## Introduction

Liver is the central drug-metabolizing organ and hepatotoxicity is a major cause for drug withdrawal^1^. In pharma industry, there is an urgent need for an adequate evaluation of the potential of the drug to induce drug induced liver injury (DILI) as DILI represents an important cause of morbidity and mortality^2^. To pursue drug metabolism studies an easily scalable model to study libraries of slowly metabolizing drug candidates is needed. Current practices to assess DILI include animal models that are expensive, non-human and ethically questionable as well as two-dimensional (2D) cultures of human hepatocytes and hepatocyte co-cultures with nonparenchymal cells that involve a complicated experiment setup^2^. 2D cultures are suitable for initial assessment of drug metabolism, however, their disadvantage lies in their short-term nature as hepatocytes dedifferentiate into nonfunctional cells within a few days in the standard culture conditions^3^. In addition, 2D cultured cells lack cell-extracellular matrix contact, intercellular communication and spatial heterogeneity that are essential for maintaining long-term liver structure and functions^4^. Three-dimensional (3D) culture models facilitate the maintenance of a similar tissue architecture to that occurring in in vivo situation, which supports correct cell polarization and differentiation status. In recent years several 3D hepatocyte culture and co-culture methods have been described^5^, which include both non-scaffold-based methods utilizing 3D liver spheroids^6^ (hanging drop liver spheroids^7^, spheroids grown on ultra-low attachment microplates^8^, microfluidic 3D cell culture^9,10^) and scaffold-based methods (3D hydrogels^11^, polymeric hard scaffolds^12^, biologic natural scaffolds^13^, micropatterned surface microplates^14^). More recent techniques in hepatocyte culture include 3D liver microtissue biochips coupled to a microfluidic device^15^ and bioprinting^16,17^. Despite of these advances there is a continuous need for a cost-efficient, simple and reliable long-term 3D culture for primary human hepatocytes to assess DILI. Currently the most commonly used long term hepatocyte model system is a pseudo 3D sandwich culture where the hepatocytes are cultured in 2D conditions between two layers of collagen^18^ or collagen and Matrigel^19^. However, this model is time consuming and costly to set up, and the results may vary due to the batch-to-batch variation of the culture reagents.

It is known that artificially generated natural protein-based matrices may mimic several aspects of the native environment. Gelatin is a physiological non-synthetic material that consists of a mix of collagens—the main constituents of the extracellular matrix (ECM) of animal tissues and is prepared from natural sources with minimal processing. Furthermore, gelatin forms an excellent base for creating semi-artificial materials that mimic natural ECM as this has similar tensile strength to natural ECM when cross-linked in a suitable manner and it is possible to enrich the gelatin-derived structures with other relevant proteins to enhance the homing and differentiation status of the contained cells^5^. Whereas a wide range of methods have been applied to design fibrous gelatin-based preforms for tissue engineering, electrospinning stands out as the most widely used approach. In an earlier work we have characterized a thermally cross-linked glucose-containing electrospun gelatin material that was suitable for growing primary human fibroblasts^20^. However, we^20^ and other research groups have shown that electrospinning typically produces nonwovens of in-plane oriented fibers that limit cell migration into deeper layers resulting in culture constructs that are essentially 2D and as such not suitable for modelling of parenchymal organs such as liver^21–23^. In recent years, solution blow spinning (SBS) has emerged as alternative method that can produce three-dimensional nano- and microfiber structures at higher throughput using a significantly simpler and less specific setup^24,25^. SBS has been successfully applied for creating 3D structures from fish gelatin; however, the single published attempt to utilize mammalian (porcine) gelatin as the input was unsuccessful^26^.

In the present work we, to our best knowledge, for the first time successfully applied the SBS method and glucose-enhanced thermal crosslinking to produce 3D scaffolds from mammalian (porcine) gelatin that faithfully mimic main aspects of natural extracellular collagen matrix. Furthermore, we used these 3D scaffolds as culturing substrates for human primary hepatocytes to create 3D human liver tissue equivalents that maintained the characteristic differentiation status and main physiological properties of these cells in long term cultures. We conclude that combining the SBS-generated mammalian gelatin scaffolds with human primary hepatocytes can be used for creation of a simple and efficient platform for in vitro research and drug testing applications related to human liver.

## Materials and methods

### Generation of gelatin-based mats

Gelatin (type A from porcine skin, 80 Bloom), d-( +)-glucose and glacial acetic acid were purchased from Sigma-Aldrich. Gelatin was dissolved in 10 M aqueous acetic acid solution at about 40 °C by vigorous stirring to obtain solutions containing 20% gelatin. Glucose was then added to the mixture in 1:6 ratio to gelatin. Obtained solution was then transferred to a commercial Tamiya airbrush (HG, WideTrigger, 0.5 mm nozzle, 15 ml). Compressed air from a central line in the building was adjusted to 2 bar pressure using a generic valve and fed into the airbrush with the gas inlet and nozzle in fully open positions. The airbrush was held in hand and the fibers were collected on a nylon mesh fabric on a 40 × 40 cm frame and placed at 70 distance under a fume hood. Systematic trial and error experiments were conducted to reach the described setup that produced fairly uniform fiber material with about 300 µm thickness over several cm^2^ in the middle. The deposited material was removed from the collector fabric and heated at in an oven at 175 °C for 1 h This time is sufficient for cross linking to occur and does not cause gelatine melting or decomposition. 7 mm round punch was used to cut round pieces from the mat to fit the wells in the 96-well tissue culture plate (Corning Incorporated, Kennebunk, ME) used in cell culture studies. The fiber samples were fixed to the bottom of the wells by placing simple polypropylene ribbon as spring adaptors on their edges.

### Enrichment of mats

Prior cell culture the mats were enriched for 3 h at 4 °C with either Matrigel Matrix (20 × dilution, Corning), laminin-111 (concentration 25 µg/ml, BioLamina) or vitronectin (concentration 12.5 µg/ml, Sigma-Aldrich) in hepatocyte culture medium. Control mats were incubated in culture medium alone. After the incubation the mats were briefly washed with phosphate buffered saline (PBS) pH 7.4 before seeding the cells.

### Scanning electron microscopy (SEM)

To simulate the behaviour of the SBS generated mats in an aqueous environment, a separate sample was prepared by soaking in water for 24 h and subsequent drying by critical point drying (Leica EM CPD300). Scanning electron microscope (Tescan Vega) was used to visualize the microstructural features of samples that were prior coated with 5 nm gold layer by Polaron SC7640 sputter coater. The images were obtained with Vega TC software. Fiber thickness was estimated from the captured images using ImageJ/FIJI software (National Institutes of Health).

### Hep G2 cell culture

Hep G2 cells were a kind gift from Dr Reet Kurg (Institute of Technology, University of Tartu). Approximately 1 × 10^5^ cells were seeded into each well of 96-well plate containing a piece of mat in 100 µl Iscove's Modified Dulbecco's Medium containing 10% fetal bovine serum (both Corning) and 1% penicillin/streptomycin (all Gibco, Invitrogen). Cells were cultured for 3 days with daily media changes. Next, the mats were processed for immunofluorescence microscopy as described below.

### Cell viability assay

Cell viability and cytocompatibility assessment were carried out using CellTiter-Glo^®^ 3D Cell Viability Assay (Promega) according to the manufacturer’s instructions.

### Human primary hepatocyte culture

Human cryopreserved hepatocytes (Corning, Discovery Labware, Woburn, MA, U.S.) were cultured in serum-free hepatocyte defined growth medium (Corning), containing epidermal growth factor (10 ng/ml), 2 mM l-glutamine (Sigma-Aldrich) and 1% penicillin/streptomycin (Gibco, Invitrogen). For long-term experiments the cells were cultured for up to 24 days with media changes every second day. Approximately 2 × 10^5^ cells were seeded into each well of 96-well plate containing a mat or on collagen I coated 24-well plates (Corning) which were overlaid with Matrigel (MG) Matrix (Corning) according to manufacturer’s instructions. The percentage of live, matrix-attached cells in culture was estimated by counting the number of detached cells at every medium renewal and at final time-point. Additionally, CellTiter-Glo 3D Cell Viability Assay (Promega) was performed to evaluate the adequacy of detached cell counting for assessing live cell numbers.

### Cytochrome P450 (CYP) induction

CYP inducibility in cultured hepatocytes was assessed as described previously^27^. Briefly, 20 days after seeding the hepatocytes 50 µM omeprazole, 20 µM rifampicin or similar volume of dimethyl sulfoxide solvent (all Sigma-Aldrich) were added to hepatocyte culture medium for 4 days with daily media replacement.

### CYP1A2, CYP2B6 and CYP3A4 activity measurements

CYP1A2, CYP2B6 and CYP3A4 activity was measured by using P450-Glo™ CYP1A2 (#V8421), CYP2B6 (#V8321) and CYP3A4 (#V9001) Assay systems (Promega) according to manufacturer’s protocols. Results were calculated against the number of viable cells in the culture which was determined by using CellTiter-Glo 3D Cell Viability Assay (Promega) according to manufacturer’s instructions.

### RNA isolation

For RNA extraction hepatocytes were lysed in 500 µl Trizol (Gibco) by repetitive pipetting. 100 µl chloroform was added to the lysates and the mixture was incubated 2–3 min at room temperature and centrifuged at 12,000*g* for 10 min at 16 °C. Following centrifugation, a colourless aqueous upper phase was transferred into a gDNA Eliminator spin column from RNeasy Plus Mini Kit (Qiagen) and RNA was further isolated with the same RNA purification kit according to the manufacturer’s protocol.

### cDNA synthesis

Total RNA was reverse transcribed with High Capacity cDNA Reverse Transcription Kit (Thermo Fisher Scientific) according to manufacturer’s instructions.

### RT-qPCR

*CYP1A2*, *CYP2B6* and *CYP3A4* qPCR was performed according to manufacturer’s protocols by using TaqMan Universal PCR Master Mix (Applied Biosystems) and FAM-MGB Taqman probes directed against *CYP1A2* (Thermo Fisher #Hs00167927), *CYP2B6* (Thermo Fisher #Hs03044634), *CYP3A4* (Thermo Fisher #Hs00604506) and *HPRT1* (Thermo Fisher #Hs02800695) as a loading control. *HNF4a*, *Vimentin* and *RPLP0* qPCR was performed by using Maxima SYBR Green/ROX qPCR Master Mix reagents (Thermo Fisher Scientific) with the following primers: *HNF4a* forw TGGTGGACAAAGACAAGAGGAAC, rev GAGCGCATTGATGGAGGGCA, *Vimentin* forw GACCAGCTAACCAACGACAAAG, rev GGTGTTTTCGGCTTCCTCTCT and *RPLP0* forw CTGGAGGGTGTCCGCAATGT, rev AGCAGCCACAAAGGCAGATGGAT. All qPCR reactions were carried out with a LightCycler^®^ 480 Instrument II (Roche) and the data acquired were analysed with LightCycler^®^ 480 Software (Roche). The results were calculated by using the ΔΔCT method against *HPRT1* or *RPLP0* as a loading control. The qPCR reactions were performed at least 3 times for each sample.

### Immunofluorescence microscopy

Mats were fixed in 4% paraformaldehyde for 10 min and permeabilized in 0.2% Triton X-100 in PBS solution for 15 min. After blocking with 5% normal donkey serum (Sigma-Aldrich) for 60 min, the slides were incubated with primary antibodies overnight at 4 °C, followed by incubation with secondary antibodies for 1 h at room temperature. Where indicated, the actin filaments were stained with phalloidin-Alexa568 (Molecular Probes, Invitrogen). Nuclei were counterstained with DAPI (diamidino-2-phenylindole, 0.1 µg/ml, Sigma-Aldrich). Used antibodies are listed in Supplementary Table S1. Images were obtained with LSM 710 confocal laser scanning microscope (Zeiss) with AlphaPlnAPO 63x/1.46 objective (Zeiss) and Orca-R2 C10600-10B camera (Hamamatsu) and analysed with ZEN 3.0 Black edition (Carl Zeiss Microscopy GmbH). The fluorescence intensity was quantified using ImageJ/FIJI software (National Institutes of Health). The level of CTCF (Corrected total cell fluorescence) was calculated using the following formula, with the data from confocal microscopy images, using: CTCF = Integrated Density – (Area of selected cell × Mean fluorescence of background readings). The fluorescence of 80–130 cells in each sample was quantified. For quantitative cell morphology analysis the long (x) and short axis (z) of cells were measured from 3D stacks using ImageJ/FIJI software.

### Urea and albumin measurements

Human primary hepatocytes were cultured in serum-free hepatocyte defined growth medium as described in section “Human primary hepatocyte culture”. Hepatocyte culture media was collected at indicated time points and stored at -20 °C before analysis. The concentration of urea was measured using urea assay kit and the concentration of albumin was assessed using BCG (Bromocresol Green) albumin assay kit (both Sigma-Aldrich) according to manufacturer’s instructions.

### Data analysis and statistics

Statistical significance was determined by one-way ANOVA followed by Turkey’s post-test (multiple comparisons) or Student’s t-test (two groups). *P* < 0.05 was considered significant.

## Results

### The microstructure of SBS gelatin mats

**Figure 1:**
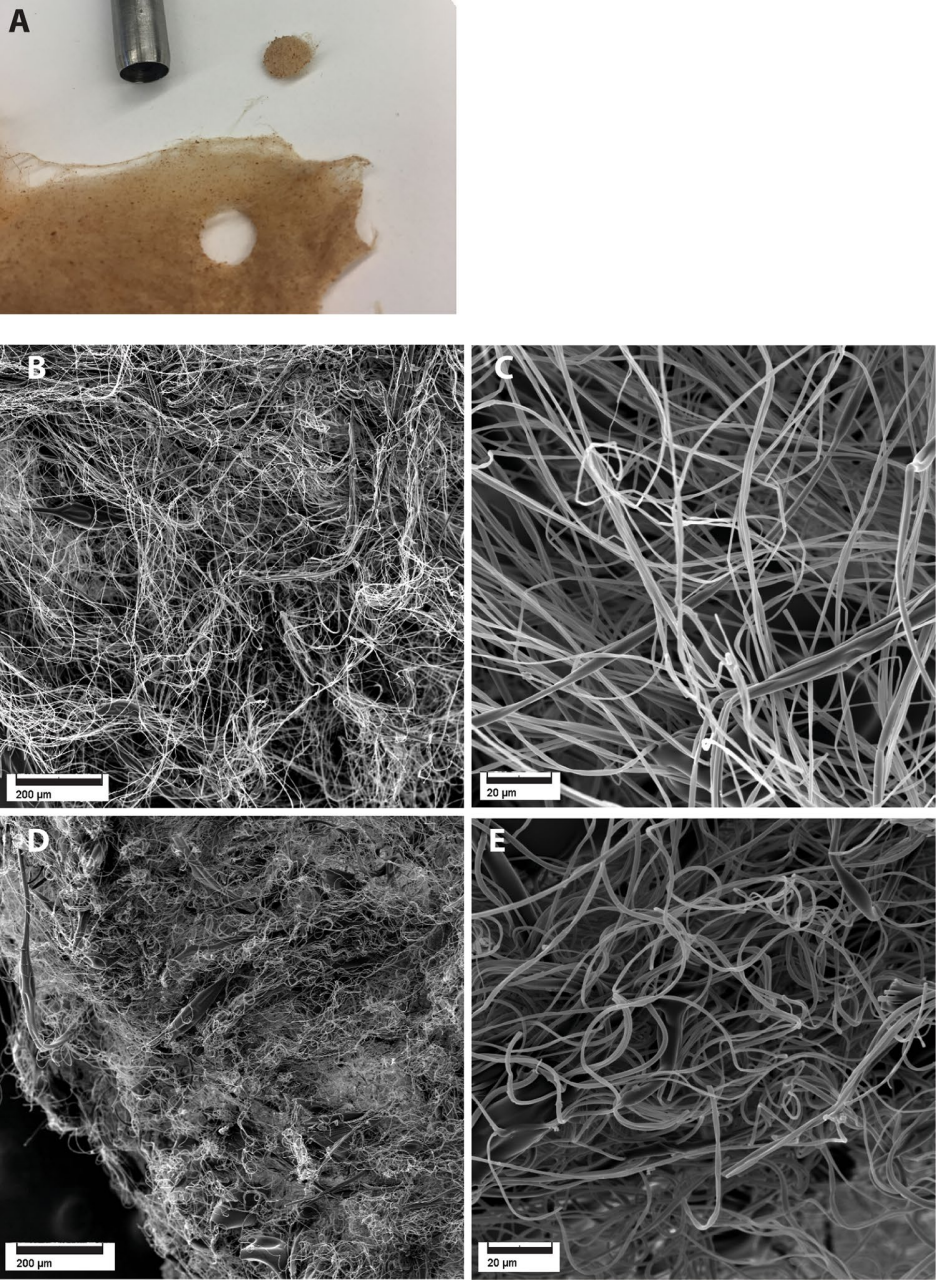
The microstructure of gelatin-based SBS mats. (**A**) Photograph of thermally treated gelatin-based mat. A disc with 7 mm diameter that was used for 3D cell culturing is shown in the top part of the panel along with the corresponding punch. (**B,C**) SEM micrographs of dry gelatin-based SBS mat obtained after thermal cross-linking. (**D,E**) SEM micrographs of SBS mats after soaking in water and subsequent drying using supercritical CO_2_. Scale bars are 200 µm on left panels and 20 µm on right panels.

The mats produced by SBS had a flocculent, cotton tuft-like appearance (Fig. 1A), suggesting a 3D fiber topology in contrast to typical 2D electrospun fiber mats^22,24^. SEM analysis of dry mats demonstrated the placement of collagen fibers in wavy bundles that were orientated in all three spatial dimensions confirming the presence of an ECM-like 3D structure (Fig. 1B,C). As thermally crosslinked glucose-gelatin is hygroscopic, the material was anticipated to swell when hydrated and we asked whether the properties of the crosslinked gelatin mats in wet environment (such as cell culture) would differ from those in the dry state. Indeed, the SEM analysis of a water-soaked mats demonstrated that in hydrated state the material was considerably denser and the voids between the fibers were shrunk. These changes, however, did not alter the overall structure of crosslinked gelatin SBS mats and the fibrous 3D nature was well preserved (Fig. 1D,E). Comparing the SBS-produced fiber mats to a natural decellularized mouse liver ECM using SEM (Supplementary Fig. S1A-B) revealed that the microarchitecture and 3D fiber arrangement of the SBS mats showed considerable degree of similarity. However, the fibers of our SBS gelatin mats were significantly thicker than those in the natural liver ECM. When the fibers in SBS mats had a diameter of 1.52 ± 0.54 µm, the decellularized liver microstructure was denser and the matrix fibers were considerably thinner with a diameter of 19.6 ± 7 nm (Supplementary Fig. S1C).

### SBS mats are cytocompatible and allow 3D cell growth

**Figure 2:**
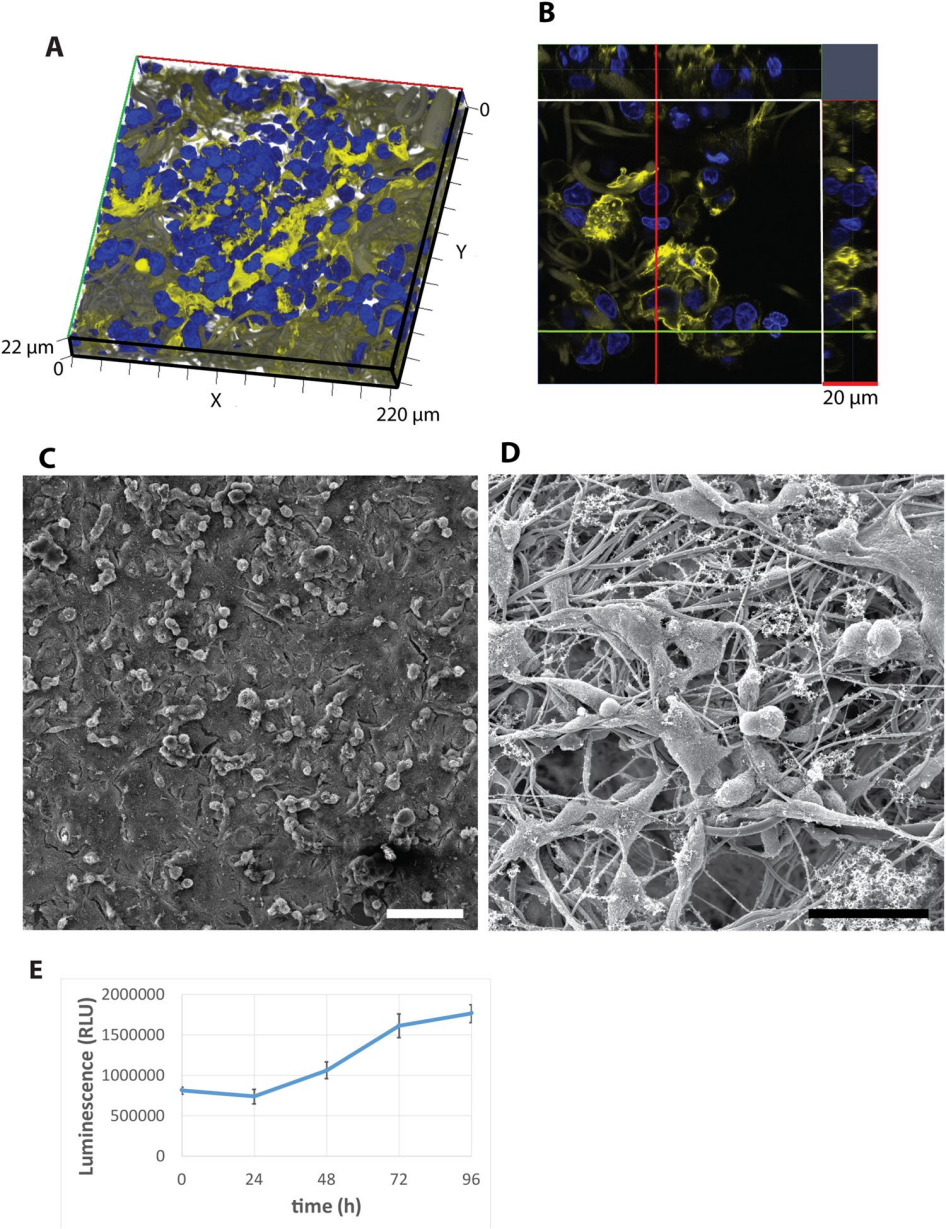
Hep G2 cells cultured on SBS mats for 96 h. (**A**) 3D reconstruction showing the Hep G2 cells grown on a SBS mat. (**B**) Ortho-view of the Hep G2 cells grown on a SBS mat. Actin cytoskeleton of cells was visualized by phalloidin staining (yellow), cell nuclei were stained with DAPI (blue). (**C,D**) SEM micrographs at different magnifications of the surface of mats populated with Hep G2 cells. Scale bars are 100 µm (**B**) and 50 µm (**C**). (**E**) Cell viability assay of Hep G2 cells cultured on SBS mats for 96 h. *RLU* relative light units. Hep G2 cells were cultured in IMDM medium containing fetal bovine serum.

To study the cytocompatibility of the mats we cultured immortalized hepatocyte cell line Hep G2 in cell culture medium containing fetal bovine serum on discs cut out from SBS mats that were fit into the wells of a 96-well tissue culture plate (Fig. 2). Cell morphology was evaluated by actin cytoskeleton staining. The cells showed normal morphology and were spread in between and around the matrix fibers. The cells formed a thick layer of pseudotissue in the 3D mat that consisted of tightly packed organoid-like structures that spanned 2–3 cell layers in depth (Fig. 2A,B). SEM of SBS mats cultured with Hep G2 cells showed fibrous matrix densely populated with cells (Fig. 2C,D). The viability of the cells on SBS mats was not reduced over time as no cytotoxicity was observed (Fig. 2E). On the contrary, the approximately two-times increase in the signal strength reflected the doubling of the cell numbers grown on SBS mats during the 96-h time window and demonstrated that the HepG2 cells were not just surviving but also proliferating on the SBS-mats (Fig. 2E).

As natural ECM is a complex structure that contains several different structural and regulative proteins, we asked whether enrichment of the processed SBS gelatin mats with common active constituents of tissue ECM—laminin, vitronectin, or a mixture of basal membrane components (Matrigel, MG) would modulate cell growth. We cultured Hep G2 cells on either uncoated SBS mats or mats preincubated with MG, laminin-111 or vitronectin solution in tissue culture media (Supplementary Fig. S2). No significant changes in cell morphology or viability were detected suggesting that uncoated 3D gelatin mats provide adequate mechanical support and homing potential for the maintenance and growth of immortalized hepatocyte-like cells.

### The mats are a suitable substrate for 3D growth of primary hepatocytes

**Figure 3:**
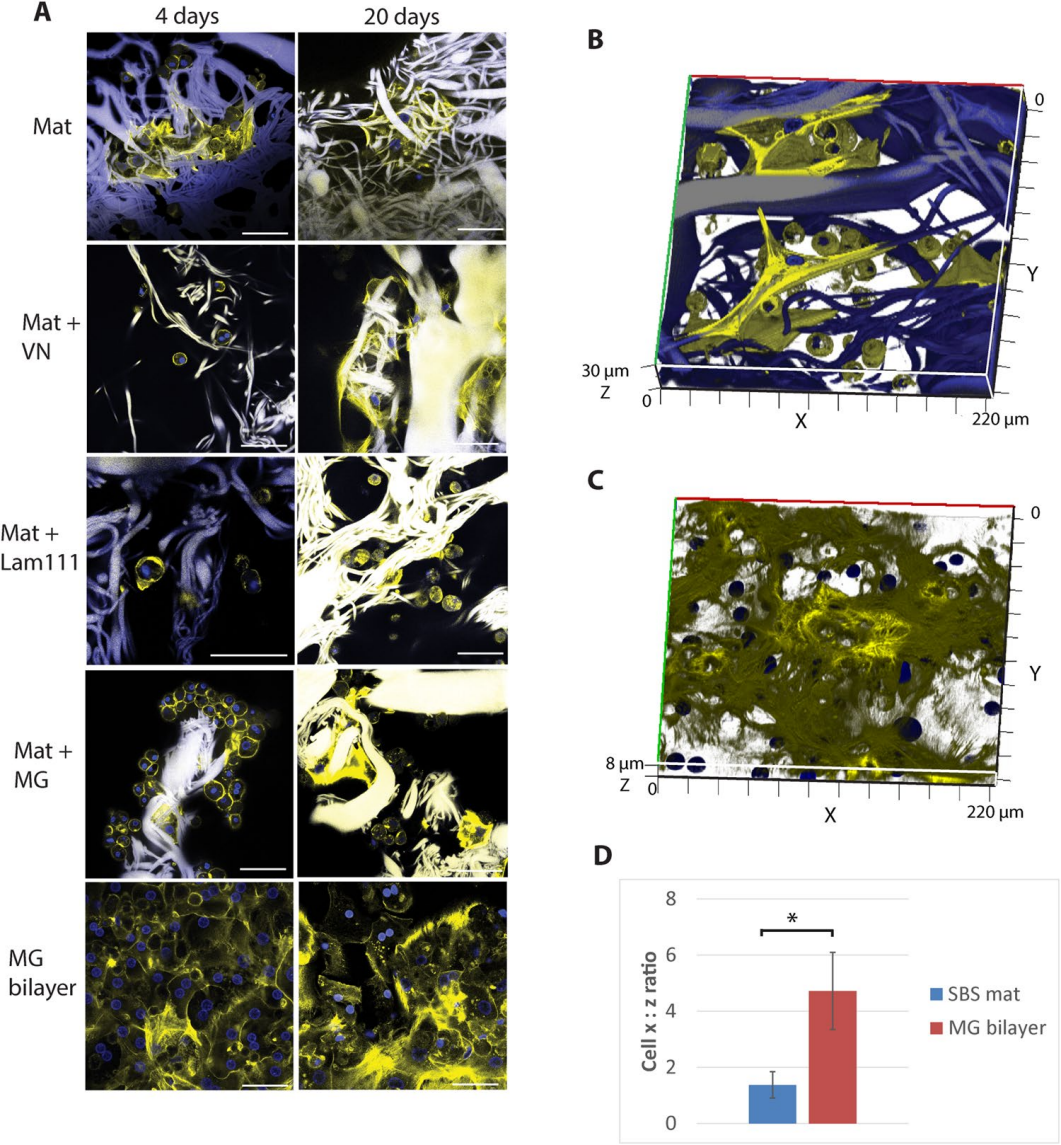
(**A**) Hepatocytes grown on SBS mats or in MG bilayer for 4 days (left panels) or 20 days (right panels). Scale bars 50 µm. 3D reconstruction of hepatocytes grown on mats (**B**) and MG bilayer (**C**) after 4 days of culture. Actin cytoskeleton of cells was visualized by phalloidin staining (yellow), cell nuclei were stained with DAPI (blue). The gelatin fibers appear as yellow or blue streaks due autofluorescence. (**D**) Quantification of cell shape using the cell length (x) to depth (z) ratio (x:z ratio). Summarized results from 3 independent hepatocyte culture replicates are presented, * indicates a statistically significant (P < 0.05) difference. Primary hepatocytes were cultured in serum-free hepatocyte defined growth medium.

To study whether the SBS mats would also support the growth of primary human liver cells we cultured human cryopreserved hepatocytes in serum-free hepatocyte growth medium on either SBS mats alone or mats enriched with MG, laminin-111 or vitronectin (Fig. 3A). As a comparison, the most commonly used primary hepatocyte culture method, collagen—MG bilayer culture, was used. The cells were cultured for 4 or 20 days and the SBS mats were studied using a confocal microscope. Similarly, to the immortalized Hep G2 cells, the primary human hepatocytes spread on and around the matrix fibers, spanning up to cell 3 layers in depth (Fig. 3B, Supplementary Fig. S3A). Cells grown in MG bilayer were flatter when compared to cells grown on SBS mats and grew as a monolayer (Fig. 3C, Supplementary Fig. S3B). To assess the cell shape differences in more detail we measured the cell length (x-axis) and cell depth (z-axis) from the z-series data from the confocal microscopy images. SBS mat-cultured hepatocytes appeared to have greater depth in the z-dimension and shorter length compared to hepatocytes in MG bilayers. Here the x : z ratio was 1.4 on average indicating a rounder and more sphere-like shape (Fig. 3D). Hepatocytes in MG bilayer cultures had the average x : z ratio 4.7 that is characteristic of a flatter, anisotropic cell shape (Fig. 3D). In long-term cultures (20 days), the hepatocytes that were grown on SBS mats retained their characteristic polygonal shape (Fig. 3A). Whereas the shape of the cells grown in MG bilayer for 20 days was significantly altered, the polygonal cell shape was replaced by an elongated irregular shape and the cells appeared to have reduced viability as exemplified by cell vacuolization (Supplementary Fig. S4). Addition of laminin-111 or vitronectin to the SBS mats did not significantly alter either the growth characteristics or the cells´ morphology during long term culture (Fig. 3A).

### SBS mats allow the maintenance of hepatocyte differentiation in long-term cultures

**Figure 4:**
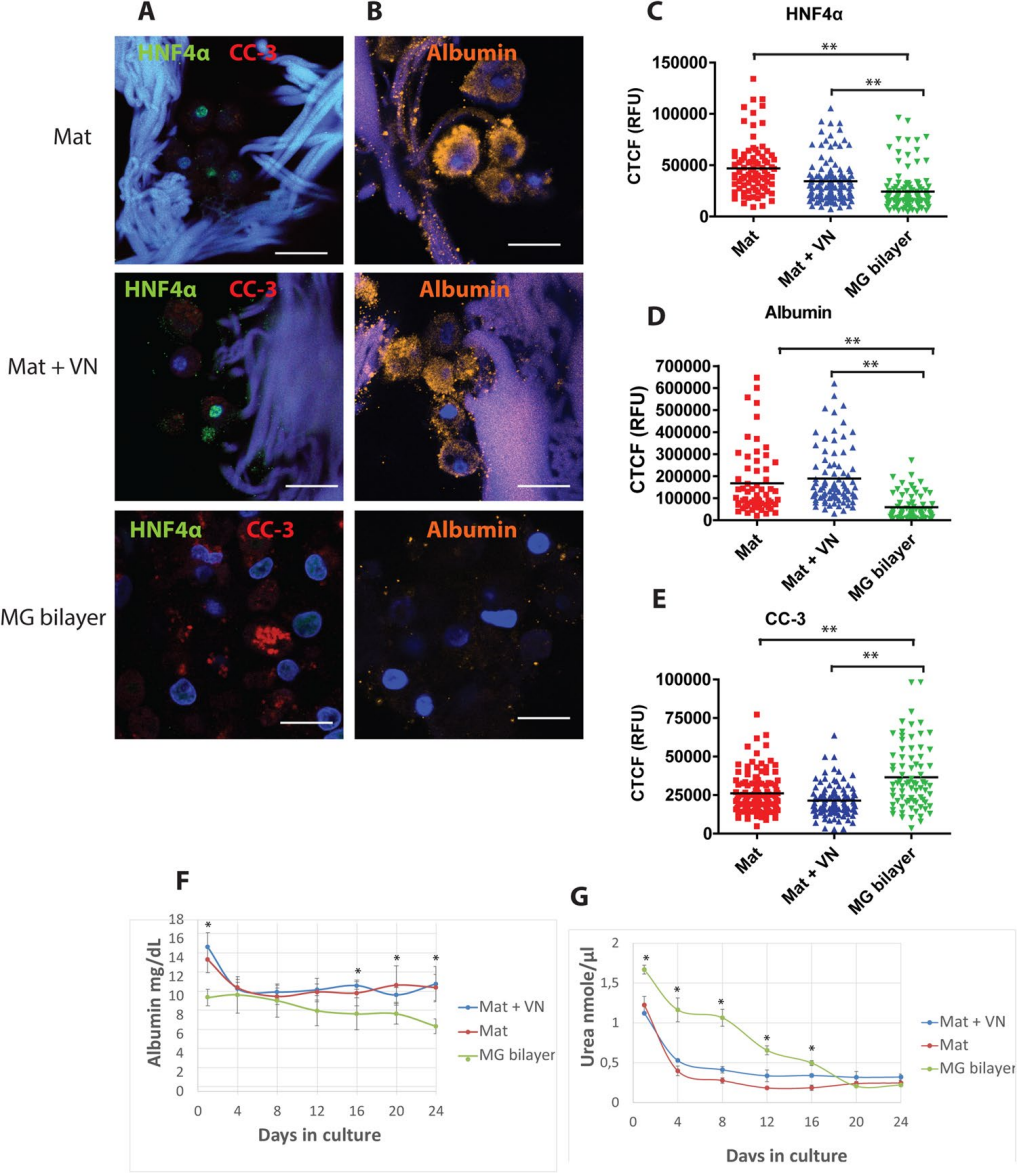
The differentiation status of primary human hepatocytes grown in long-term cultures. Confocal microscopy images (**A**) of the expression of hepatocyte-specific transcription factor HNF4α and cleaved caspase 3 (CC-3) and quantification of the corrected cell fluorescence (**C,E**). Staining of albumin in hepatocytes (**B**) and quantification (**D**). Scale bars 20 µm. The dynamics of the synthesis of hepatocyte-specific metabolites albumin (**F**) and urea (**G**) was measured in the culture media of long-term hepatocyte cultures, n = 5. * indicates a statistically significant (P < 0.05) difference, ** P < 0.001 compared to MG bilayer. Primary hepatocytes were cultured in serum-free hepatocyte defined growth medium.

Hepatocyte differentiation status during long term culturing in serum-free hepatocyte growth medium was evaluated by studying the expression of hepatocyte-specific transcription factor HNF4α and albumin. After 24 days of culture the immunofluorescence analysis of hepatocytes showed strong expression of HNF4α (Fig. 4A, Supplementary Fig. S5A) and albumin (Fig. 4B) in cells grown on SBS mats. Cells grown in MG bilayer showed significantly lower levels of HNF4α and albumin proteins (Fig. 4C,D) indicating the reduction of hepatocyte differentiation status. Interestingly, there were no significant differences between the HNF4α mRNA levels in the hepatocytes grown on SBS mats or as MG bilayer (Supplementary Fig. S5C) suggesting the existence of a posttranslational step for regulation of the HNF4α protein levels. To further examine the differentiation status of the hepatocytes, we analysed the expression of hepatocyte dedifferentiation marker vimentin^28^. Vimentin protein and mRNA expression was moderately elevated in MG bilayer culture as demonstrated by the immunofluorescence analysis (Supplementary Fig. S4A-B) and RT-qPCR (Supplementary Fig. S5D) being in line with the reduction of the HNF4α protein.

To evaluate the proportion of non-viable cells in the culture we studied the presence of cleaved caspase-3 (CC-3)—the marker of apoptosis^29^—in human primary hepatocyte long-term cultures (Fig. 4A). While the hepatocytes grown on SBS mats showed a low CC-3 signal, a large proportion of cells (in average 71%) grown in MG bilayer cultures was CC-3 positive (Fig. 4E).

To study the cell attachment and maintenance dynamics we evaluated the number of cells that remained unattached and were floating in the tissue culture media 3 h after plating and assumed that the rest of the cells were attached. Although the SBS mats had a lower cell attachment percentage (uncoated mat average 67.4%; vitronectin-coated mat 69.5%) the proportion of live matrix-attached cells remained essentially consistent from 4 to 24 days (Supplementary Fig. S6A). In comparison, the hepatocytes cultured in MG bilayer showed higher initial attachment to the 2D surface (in average 95%), however, this was followed by a fast and gradual drop in the survival rate as judged by the increase in the detached cell numbers (Supplementary Fig. S6A). To confirm the adequacy of this live cell number estimation we performed a luminescence-based cell viability assay at the end of the experiment at day 24 (Supplementary Fig. S6B-C). When we compared the calculated live cell numbers with the data obtained from the cell viability assay, we found that the numbers of cells grown on MG bilayers and 3D SBS-mats obtained by both methods did not differ significantly at the 24-day time point and concluded that our estimation of live cells in hepatocyte culture was reliable. The calculated viable cell numbers were in accordance with the measurements of two main hepatocyte metabolites—albumin and urea—from serum-free hepatocyte culture media that were consistent from 4 days onwards in cultures on SBS mats. However, the supernatants from MG bilayer cultures showed a consistent drop in the concentration of both metabolites (Fig. 4F,G). Media samples from the long term primary human hepatocyte cultures on SBS mats contained significantly more albumin (10–12 mg/dL) when compared to albumin content in the media collected from MG bilayer cultures (6–7 mg/dL) (Fig. 4F). When we normalised the albumin content to the estimated numbers of hepatocytes in culture, the differences in albumin production became even more pronounced showing approximately twofold higher albumin production per cell in hepatocytes cultured on SBS-mats compared to MG bilayer cultures at all time points (Supplementary Fig. S6D). There were no significant differences in urea concentrations at 24 days (Fig. 4G). Interestingly, we found that coating the SBS mats with vitronectin may somewhat improve the urea production per cell when compared to uncoated SBS mats (Supplementary Fig. S6E). Addition of laminin-111, vitronectin or MG-coating to the SBS mats did not significantly alter the production of albumin or urea in hepatocyte cultures on SBS mats (Supplementary Fig. S7).

### SBS mats support the maintenance of hepatocyte polarity in culture

**Figure 5:**
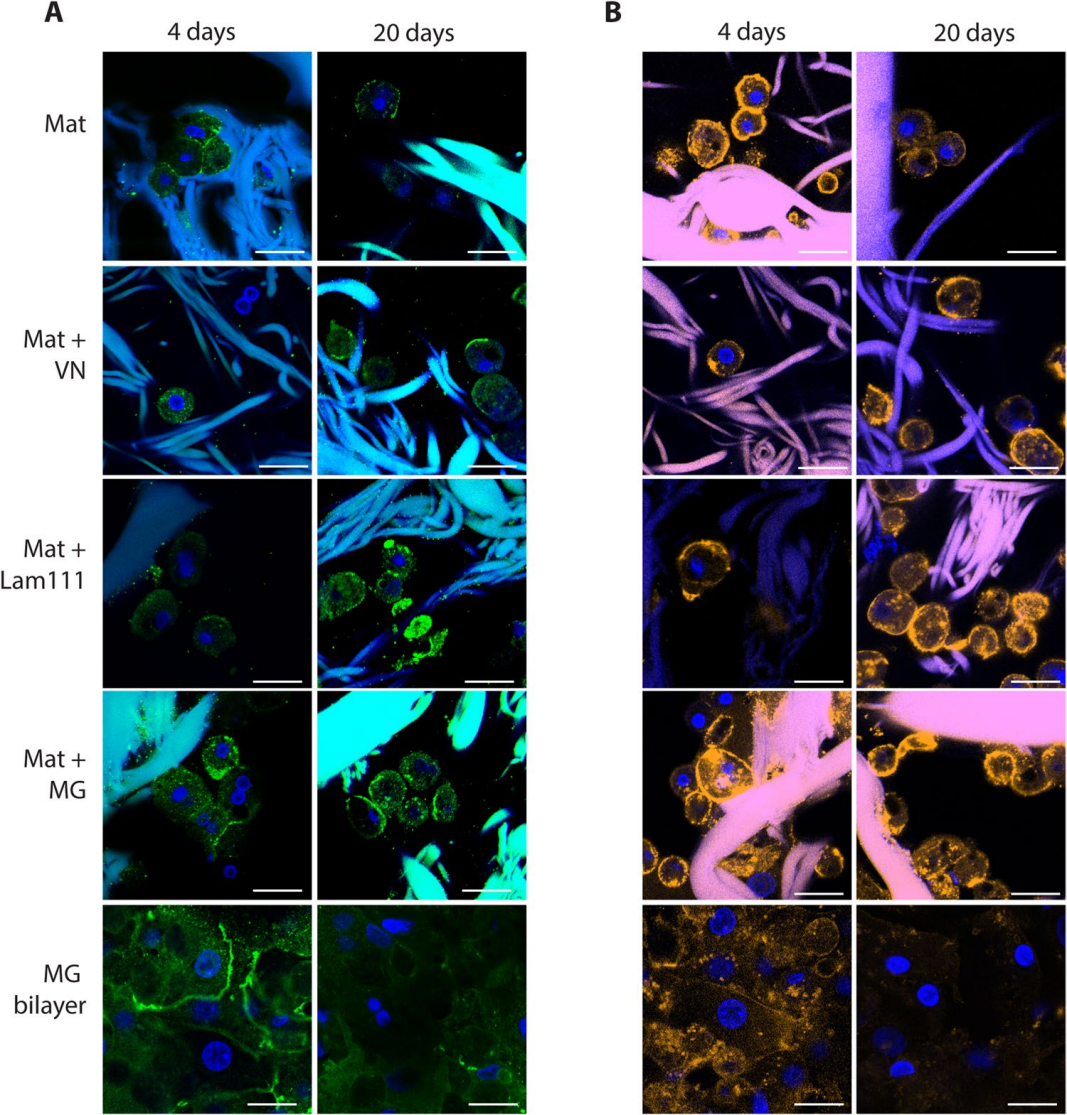
Expression of cell polarization markers in human primary hepatocytes grown on SBS mats or in MG bilayer. (**A**) Expression of tight junction component ZO1 (green) on hepatocytes cultured for 4 days (left panels) or 20 days (right panels). (**B**) Expression of transcytotic marker CD13 (orange) on hepatocytes cultured for 4 days (left panels) or 20 days (right panels). Cell nuclei were stained with DAPI (blue). Scale bars 20 µm. Primary hepatocytes were cultured in serum-free hepatocyte defined growth medium.

We examined the presence of two polarization markers, the junctional protein ZO1 and the transcytotic marker CD13 in human primary hepatocytes grown on uncoated or MG, laminin-111 or vitronectin–coated SBS mats; as a comparison we used MG bilayer culture. Immunofluorescence analysis showed that ZO1 is expressed at cell membranes at cell–cell contacts (Fig. 5A, Supplementary Fig. S8). ZO1 expression appeared consistent in short-term and long-term hepatocyte cultures on 3D mats irrespective of their coating (Fig. 5A, Supplementary Fig. S8, S9A). However, the hepatocytes grown as MG bilayer had significantly reduced ZO1 expression by day 20 in culture (Supplementary Fig. S9A). CD13 showed strong membrane localisation pattern in all hepatocytes grown on mats (Fig. 5B). Hepatocytes grown on MG bilayer showed diffuse and predominantly cytoplasmic expression of CD13 at 4 days of culture and the expression was significantly reduced by 20 days of culture (Fig. 5B, Supplementary Fig. S9B).

### SBS mats facilitate the induction of hepatocyte-specific CYP gene expression

**Figure 6:**
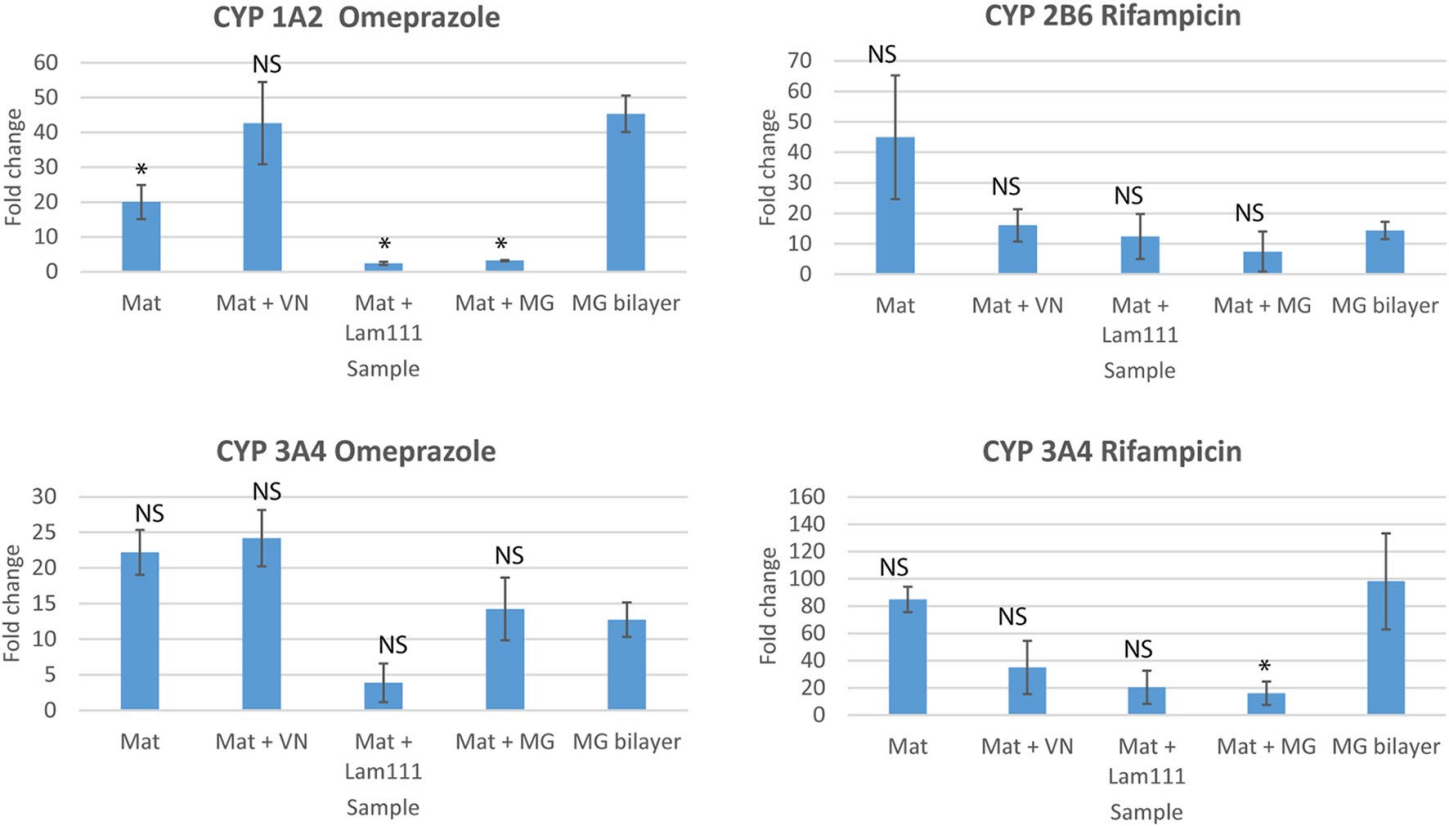
Cytochrome P450 induction in hepatocytes cultured on SBS mats or in MG bilayer. CYP1A2 and CYP3A4 were induced by omeprazole, CYP2B6 and CYP3A4 were induced by rifampicin. The human primary hepatocytes were cultured for 20 days and subsequently treated with indicated compounds for 4 days, n = 5. CYP induction was measured by RT-qPCR, fold changes in mRNA expression were calculated relative to uninduced control cells. * indicates a statistically significant (P < 0.05) difference compared to MG bilayer; *NS* not significant. Primary hepatocytes were cultured in serum-free hepatocyte defined growth medium.

Next, we tested whether the human primary hepatocytes grown on SBS mats were able to upregulate the expression of CYP1A2, CYP2B6 and CYP3A4 mRNA in response to known inducers of CYP transcription (Fig. 6). RT-qPCR showed more than a 40-fold induction of CYP1A2 mRNA in response to omeprazole treatment in cells grown on vitronectin enriched SBS mats (in average 43-fold) and on MG bilayers (in average 45-fold); a 20-fold induction of CYP1A2 in average was detected in cells grown on un-enriched SBS mats. In laminin-111 and MG-enriched SBS mats the level of CYP1A2 induction was considerably lower, only 2–3- fold in average. Omeprazole induced the increase in CYP3A4 transcription equally well in un-enriched and vitronectin-enriched SBS mats with 22-fold and 24-fold changes, respectively. In MG bilayer and MG-enriched SBS mats the increase in CYP3A4 mRNA was 13–14-fold and the lowest induction was again seen in laminin-111-enriched SBS mats with an average of fourfold change. CYP3A4 induction in response to rifampicin treatment was the highest in un-enriched SBS mats and in MG bilayer cultures with averages of 85- and 98-fold change, respectively. The CYP3A4 mRNA increase was lowest in laminin-111- (20-fold) and MG-enriched SBS mats (16-fold) with an intermediate induction in cells grown in vitronectin-enriched SBS mats (35-fold). Rifampicin-induced increase in CYP2B6 mRNA was the highest in cells grown on un-enriched SBS mats (in average 45-fold), in rest of the samples the induction of CYP2B6 mRNA remained below 20-fold, in case of vitronectin-enriched SBS mats below tenfold.

**Figure 7:**
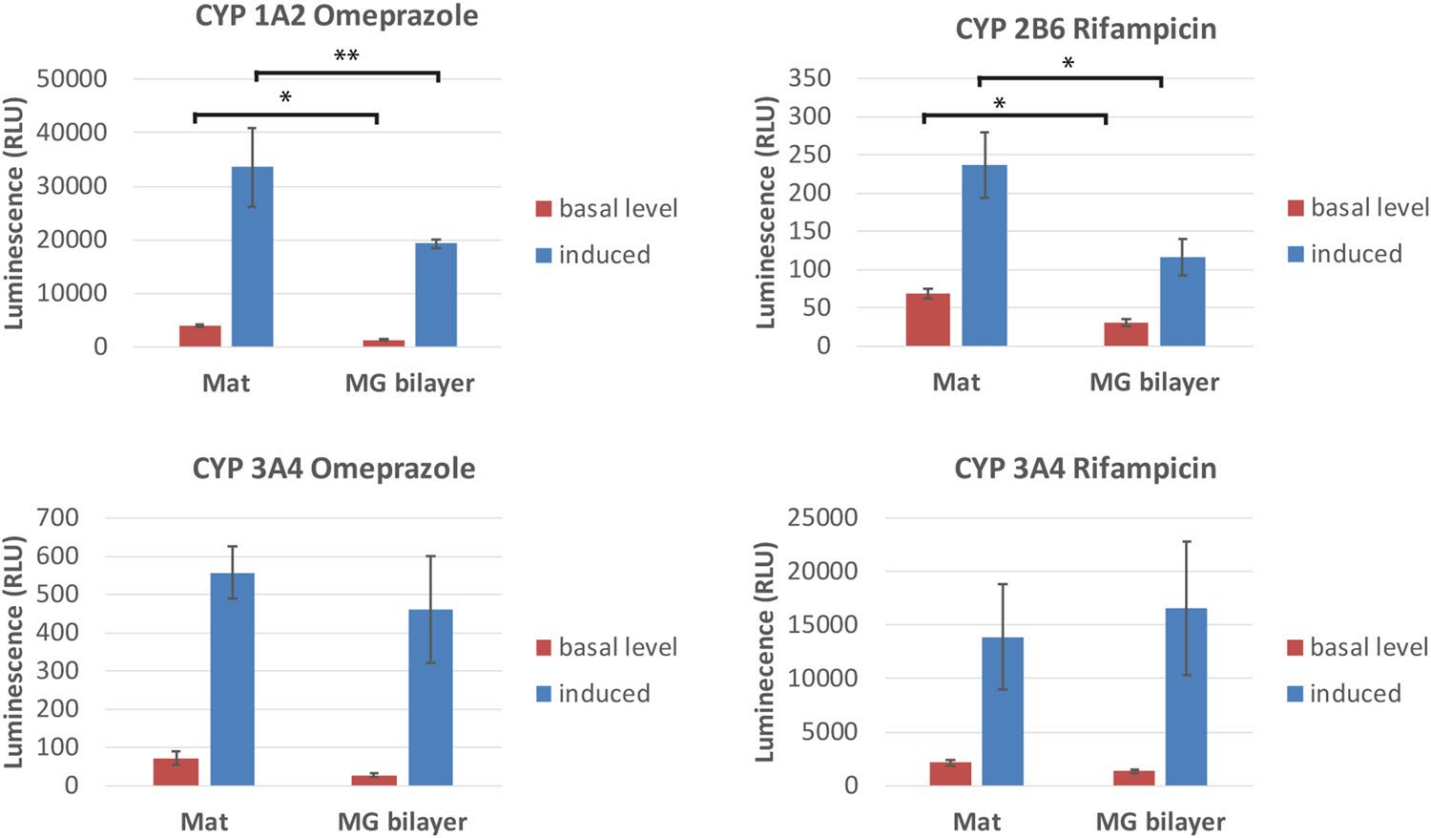
Quantification of CYP 1A2, 2B6 and 3A4 induction by enzymatic activity. CYP1A2 and CYP3A4 were induced by omeprazole, CYP2B6 and CYP3A4 were induced by rifampicin. The human primary hepatocytes were cultured for 20 days and subsequently treated with indicated compounds for 4 days, n = 5. CYP activity was quantified by luminescent method. * indicates a statistically significant (P < 0.05) difference, ** P < 0.01 compared to MG bilayer. *RLU* relative light units. Primary hepatocytes were cultured in serum-free hepatocyte defined growth medium.

To further substantiate our findings, we performed functional analysis of CYP enzyme activity of hepatocytes grown for 24 days in SBS mats or in MG bilayer (Fig. 7). We found that the enzymatic activity of CYP1A2 and 2B6 was even higher in the hepatocytes grown on SBS mats at both basal level and when induced by omeprazole or rifampicin, respectively, when compared to the hepatocytes grown in MG bilayer. The activity of CYP3A4 in hepatocytes grown in SBS mats and MG bilayer system was similar at both basal level and when induced with omeprazole or rifampicin.

## Discussion

In this work we demonstrate that the SBS method can be successfully utilized to produce 3D glucose-gelatin preforms that are excellent culturing substrates for human primary hepatocytes and enable the creation of 3D human liver tissue equivalents that recapitulate the main aspects of human functional liver tissue. This novel 3D hepatocyte culture model was shown to be superior to the most commonly used alternative to hepatocyte culture—MG-collagen bilayer—particularly in long term culture experiments where the SBS mats supported the maintenance of hepatocyte differentiation in serum-free culture medium for more than 3 weeks.

Although SBS is a very simple method for producing fine fibers from various materials, finding suitable parameters for producing continuous and uniform fibers is challenging. We used relatively long nozzle-to-collector distance for fiber production, which leaves sufficient time for the acetic acid and water to evaporate before deposition of the formed fibers. Based on our prior testing with various gelatins, we found that lower bloom gelatins are more suitable for electrospinning by SBS. Previous studies applied SBS to generate 3D nonwovens from fish gelatin while the generation of similar materials from porcine gelatin was unsuccessful^26^. Here we report, for the first time, the creation of 3D ECM-like mats from porcine gelatin by utilizing SBS method. The advantages of using porcine gelatin over the fish equivalent are its more similar composition to human collagen matrix, better availability and lower cost. An advantage of our SBS mats over natural matrices such as decellularized tissue is that the mats consist of thicker threads with increased porosity. As this artificial matrix is less dense than decellularized liver, it facilitates effective cell penetration of cells into the artificial ECM in culture conditions. Nevertheless, the microstructure of SBS mats was shown to be very similar to a decellularized liver matrix in 3D fiber orientation and microstructure. Compared to most commonly used pseudo-3D hepatocyte culture model where the cells obtain a relatively flat morphology between collagen-MG layers, the fibrous structure of SBS mats creates a more natural extracellular environment and cells can spread in 3D.

Collagens are the main components of fibrous matrices in natural ECMs; however, biological matrices contain several proteins that can have a substantial impact on cell growth and differentiation. Laminins have been studied for decades, mainly because of their role in basal membrane assembly, embryogenesis and potential utilization as a substrate for tissue engineering^30^. Secreted ECM component vitronectin can be used as a substrate to culture many types of primary cells including embryonic stem cells^31^ and we have previously shown that it accumulates in damaged human and mouse livers^32^. However, the differences between the ability to support the metabolic activity and differentiation status of primary liver cells were generally marginal as also non-enriched SBS mats without any additional components were proven to be an excellent substrate for the maintenance and growth of both immortalized and primary hepatocytes.

Hepatocyte polarity is essential for liver structure and function. In vitro studies with murine hepatocytes have indicated that cell polarity is directly correlated with the maintenance of cell differentiation^33^. Previous studies with in vitro cultured hepatocytes have shown that 2D cultured hepatocytes lose or show significantly altered expression of cell polarization markers^33^. Here we show that SBS mats support the expression of hepatocyte polarization markers tight junction component ZO1 and the transcytotic marker CD13. This was particularly evident in long term cultures where the 3D substrate was more efficient in supporting hepatocyte polarization and differentiation when compared to the most often utilized primary hepatocyte cultivating system—the MG bilayer.

Recently, 3D spheroid-based cytotoxic assays have emerged as a promising approach for DILI evaluation. A recent multicenter study showed that such systems are superior to conventional MG bilayer cultures in respect of proteome stability and displayed increased sensitivity in long-term repeated exposure experiments^34^. Although very relevant for studying hepatotoxicity such systems lack the mechanical supportive mesh that has been shown to play a major role in regulating hepatocyte behaviour as substrate stiffness has been demonstrated to be an important factor in tissue development, differentiation and disease^35–37^. The extent to which the lack of supportive structures affects the reactivity of primary hepatocytes to external stimuli is yet to be determined in future head-head comparisons.

**Figure 8:**
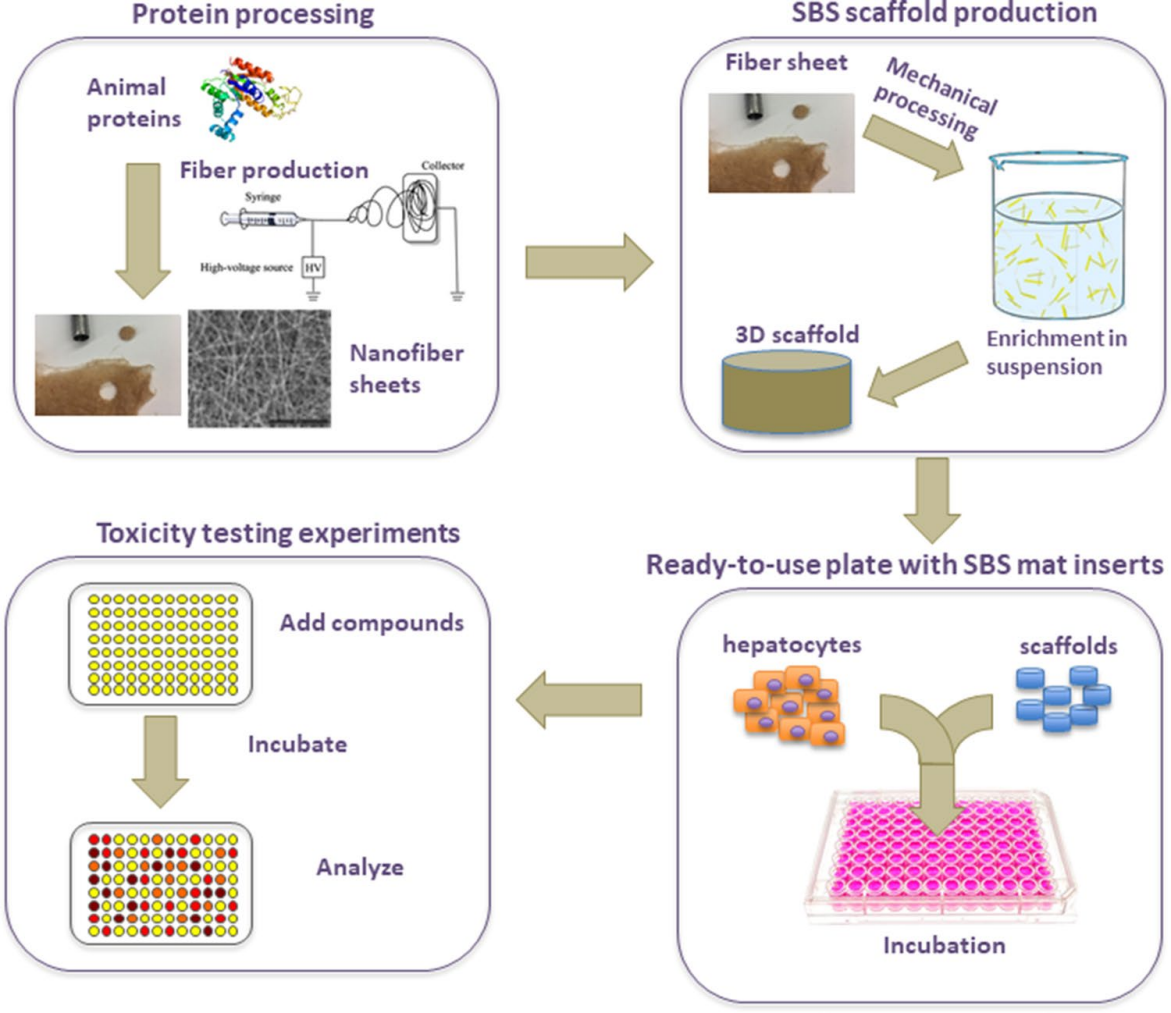
SBS mat technology summary. More details can be found in “Materials and methods” section.

In conclusion, our data suggest that the gelatin mats generated via SBS are well-suited and easy to use alternatives for long term hepatocyte drug screening studies, as the 3D substrate allows maintenance of hepatocyte-specific functions stable over long time periods. Furthermore, the mats can be easily inserted into the wells of 96-well plates, the cells in the mats are easily accessible to various chemicals including cell lysis buffers and there is no need for elimination of the MG layer prior cell lysis for further analysis. The 3D liver cultivation method is potentially more easily maintained by robotic systems and is anticipated to be well compatible with high-throughput screening methods (Fig. 8).

## Supplementary Information

Supplementary Information.

## Abbreviations

2D: 2-Dimensional
3D: 3-Dimensional
CC-3: Cleaved caspase-3
DAPI: Diamidino-2-phenylindole
DILI: Drug induced liver injury
ECM: Extracellular matrix
MG: Matrigel
PBS: Phosphate buffered saline
SBS: Solution blow spinning
SEM: Scanning electron microscopy
