# Supplementary material for: Long-term maintenance of functional primary human hepatocytes in 3D gelatin matrices produced by solution blow spinning
Source: Sci Rep. 2021 Oct 11;11:20165. doi: 10.1038/s41598-021-99659-1 (PMC8505433; doi:10.1038/s41598-021-99659-1)
Supplement: Supplementary file 1 — Supplementary Information. [file 41598_2021_99659_MOESM1_ESM.pdf]

Supplementary data

Long-term maintenance of functional primary human hepatocytes in 3D gelatin matrices produced by solution blow spinning

Mariliis Klaas[1*], Kaidi Möll[2*], Kristina Mäemets-Allas[1], Mart Loog[2], Martin Järvekülg[3] and Viljar Jaks[1,4#]

[1]Institute of Molecular and Cell Biology, University of Tartu, Riia 23b, 51010, Tartu, Estonia

[2]Institute of Technology, University of Tartu, Nooruse 1, 50411, Tartu, Estonia

[3]Institute of Physics, University of Tartu, Laboratory of Physics of Nanostructures, W. Ostwaldi 1, 50411, Tartu, Estonia

[4]Tartu University Hospital, Dermatology Clinic, Raja 31, 50417, Tartu, Estonia

[*]These authors contributed equally to this work

#To whom correspondence should be addressed: Viljar Jaks, Institute of Molecular and Cell Biology, University of Tartu, Riia 23b, 51010, Tartu, Estonia; phone: +372 737 4069; email: viljar.jaks@ut.ee.

Supplementary Methods

Liver decellularization

Decellularized liver samples from normal mouse livers were prepared as described previously[1]. Wild type CBA/J mice at 8–12 weeks of age were used in experiments. Mice were anesthetized by intraperitoneal injection of diazepam and hypnorm (1:1, 200µl). Hepatic portal vein was cannulated, and liver perfusion was carried out at 37 °C and at the speed of 5 ml/min. Mouse liver was perfused sequentially with heparinized phosphate buffered saline (PBS) (12.5U heparin/ml, AppliChem, Darmstadt, Germany) for 10min, 1% SDS and 1% Triton X-100 for 2 h each followed by perfusion with water for 30min and PBS for 2h. The procedures involving animals were carried out in accordance to the guidelines approved by the Commission of Laboratory Animal Licenses at the Estonian Ministry of Agriculture (license number 25).

SEM analysis of decellularized liver samples

The samples were dehydrated through alcohol gradient starting at 50% ethanol up to 100% ethanol. Samples were dried using a Leica EM CPD300 critical point dryer (Leica Microsystems GmbH, Wetzlar, Germany) and covered with a 5nm layer of gold using a Quorum Technologies Polaron SC7640 Sputter Coater. Fibrous structure of liver matrix was investigated by scanning electron microscope FEI Nova NanoSEM 450 (FEI, Eindhoven, the Netherlands). Fiber thickness was estimated from the captured images using ImageJ/FIJI software (National Institutes of Health).

Supplementary Tables

Table S1. Antibodies used in immunofluorescence analysis

**Antibody**	**Host, cat no**	**Dilution**	**Source, reference**
Albumin	MAB1455	1:100	R&D SYSTEMS
Alexa Flour 488<br>Donkey anti-Goat	A11055	1:1000	Thermo Fisher (Invitrogen)
Alexa Flour 488<br>Donkey anti-Rabbit	A21207	1:1000	Thermo Fisher (Invitrogen)
Alexa Flour 568<br>Donkey anti-Rabbit	A10042	1:1000	Thermo Fisher (Invitrogen)
Alexa Flour 647<br>Donkey anti-Mouse	A31571	1:1000	Thermo Fisher (Invitrogen)
CD13	Mouse, 301701	1:100	Biolegend
Cleaved Caspase-3	Rabbit, 9664 (S)	1:400	Cell Signaling
Hnf4α	Goat, sc-6556	1:200	Santa Cruz Biotechnology
ZO-1	Rabbit, 21773-1-AP	1:100	Proteintech

Supplementary Figures

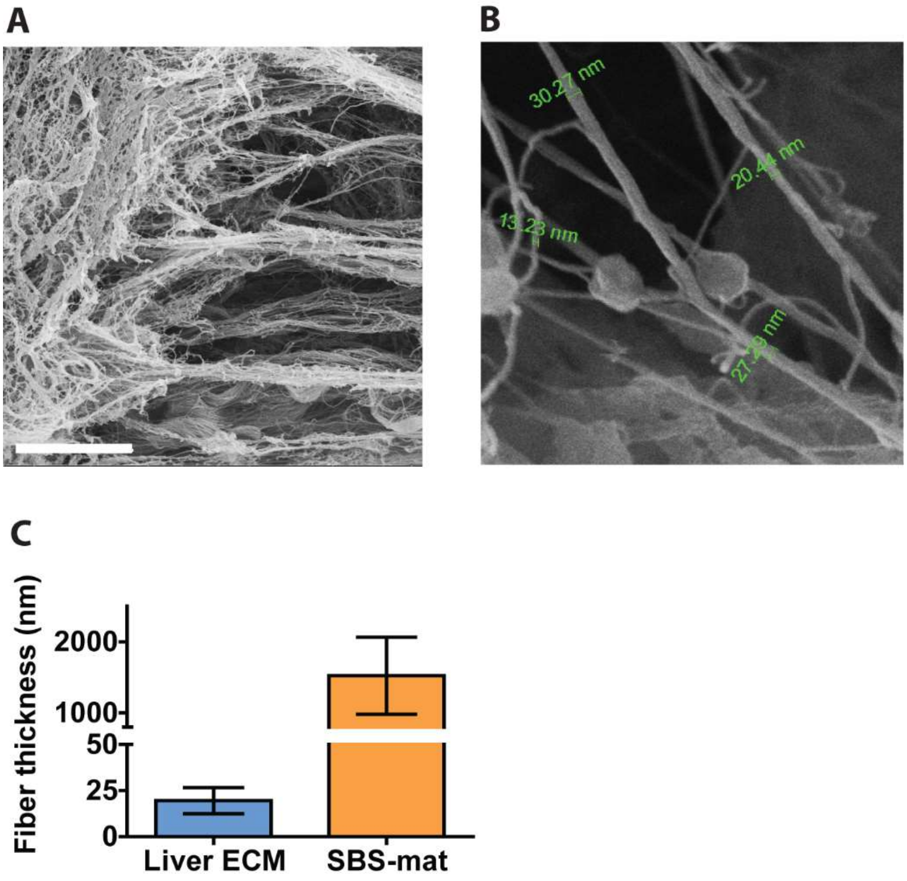

Figure S1. (**A-B**) SEM micrographs of decellularized mouse liver. (**A**) Scale bar is 20 µm; (**B**) A representative higher magnification image of liver ECM with indicated fiber thickness measurements. (**C**) Quantification of the estimated fiber thickness measurements in mouse liver ECM and SBS-mats.

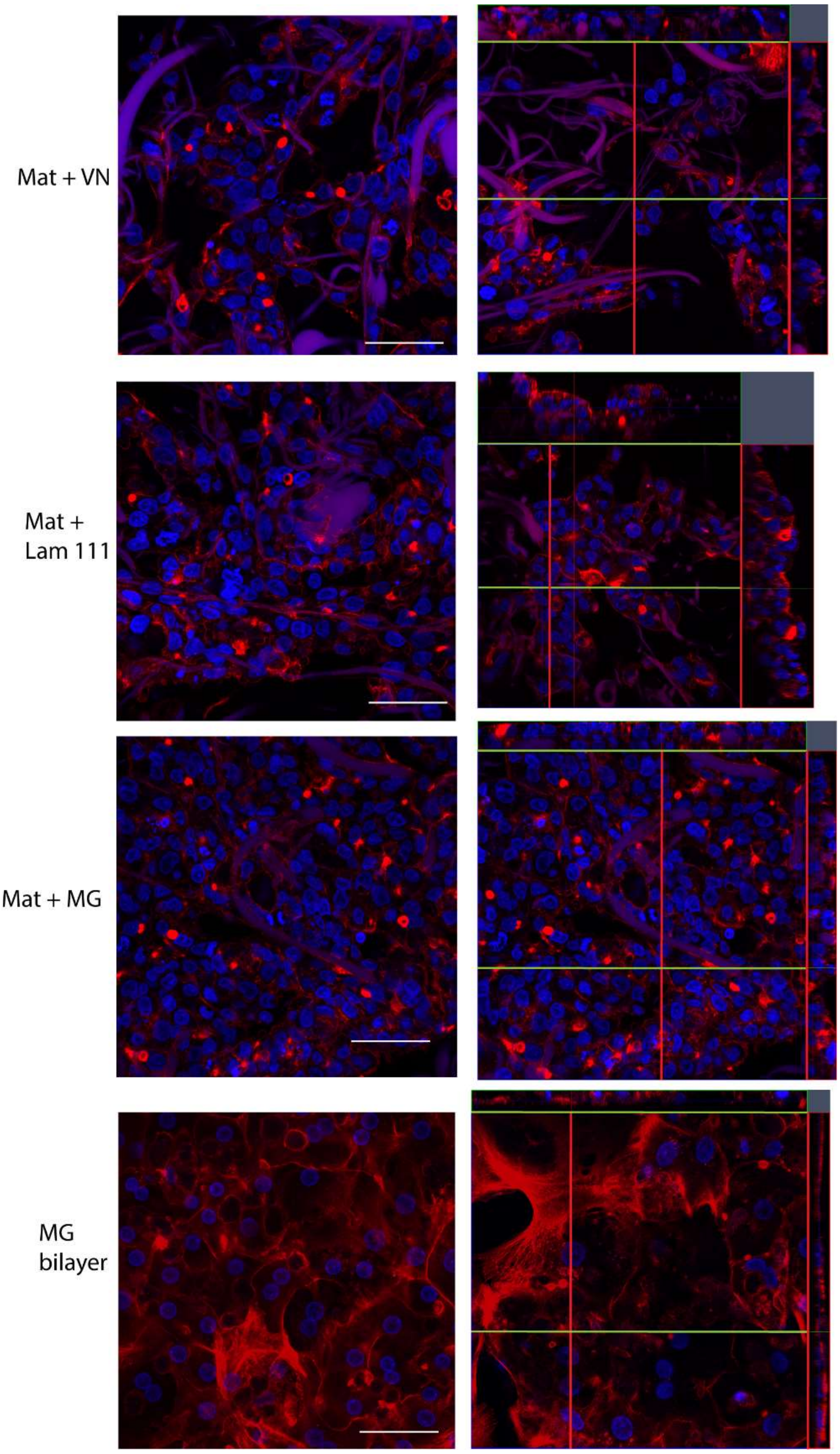

Figure S2. Confocal microscopy images of SBS mats enriched with vitronectin, laminin-111 or MG cultured with Hep G2 cells and MG bilayer. Actin cytoskeleton of cells was visualized by phalloidin staining (red), cell nuclei were stained with DAPI (blue). Scale bar is 50 µm.

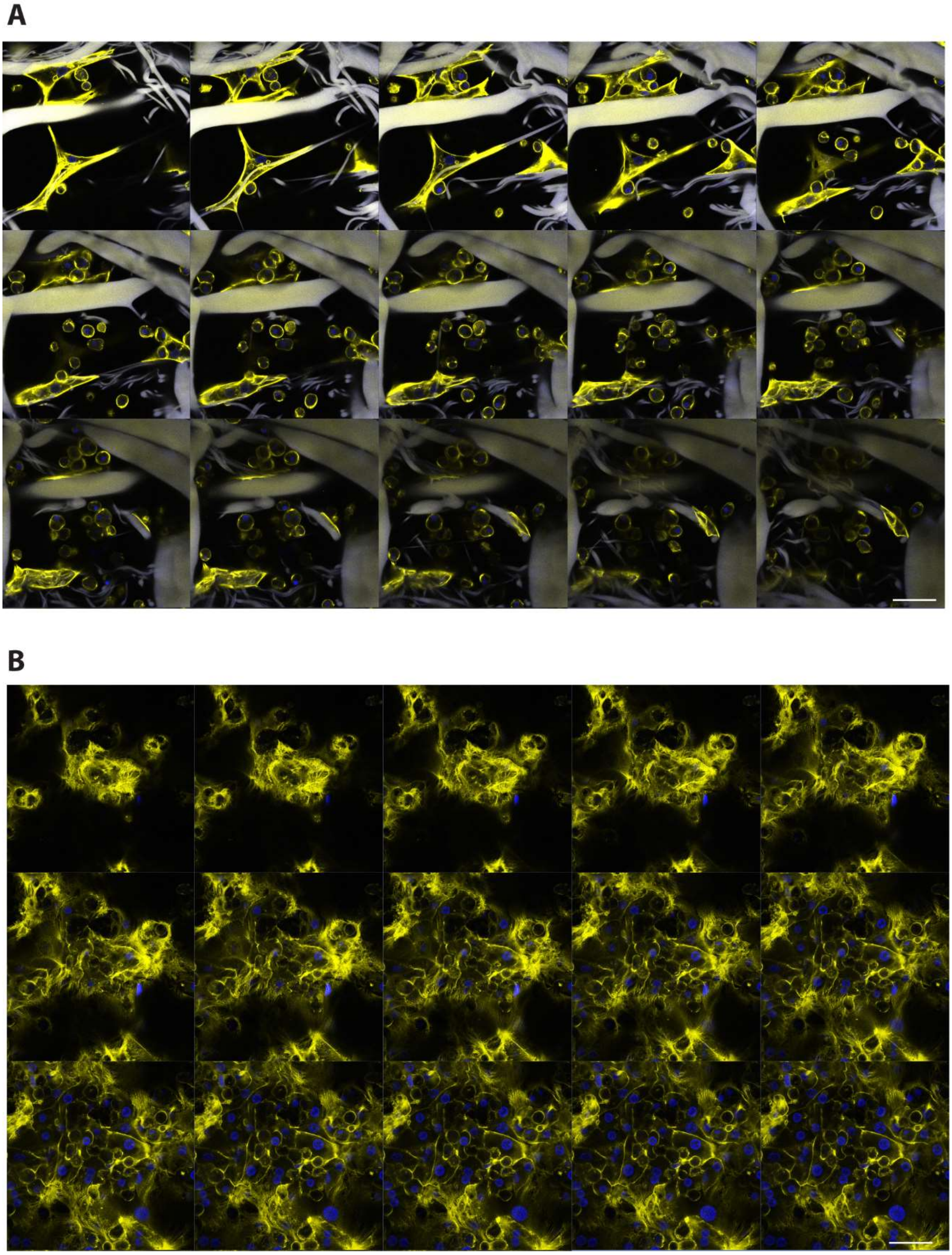

Figure S3. Series of confocal microscopy Z-stack images of SBS mats (**A**) or MG bilayer (**B**) cultured with primary human hepatocytes. Actin cytoskeleton of cells was visualized by phalloidin staining (yellow), cell nuclei were stained with DAPI (blue). Scale bars 50 µm.

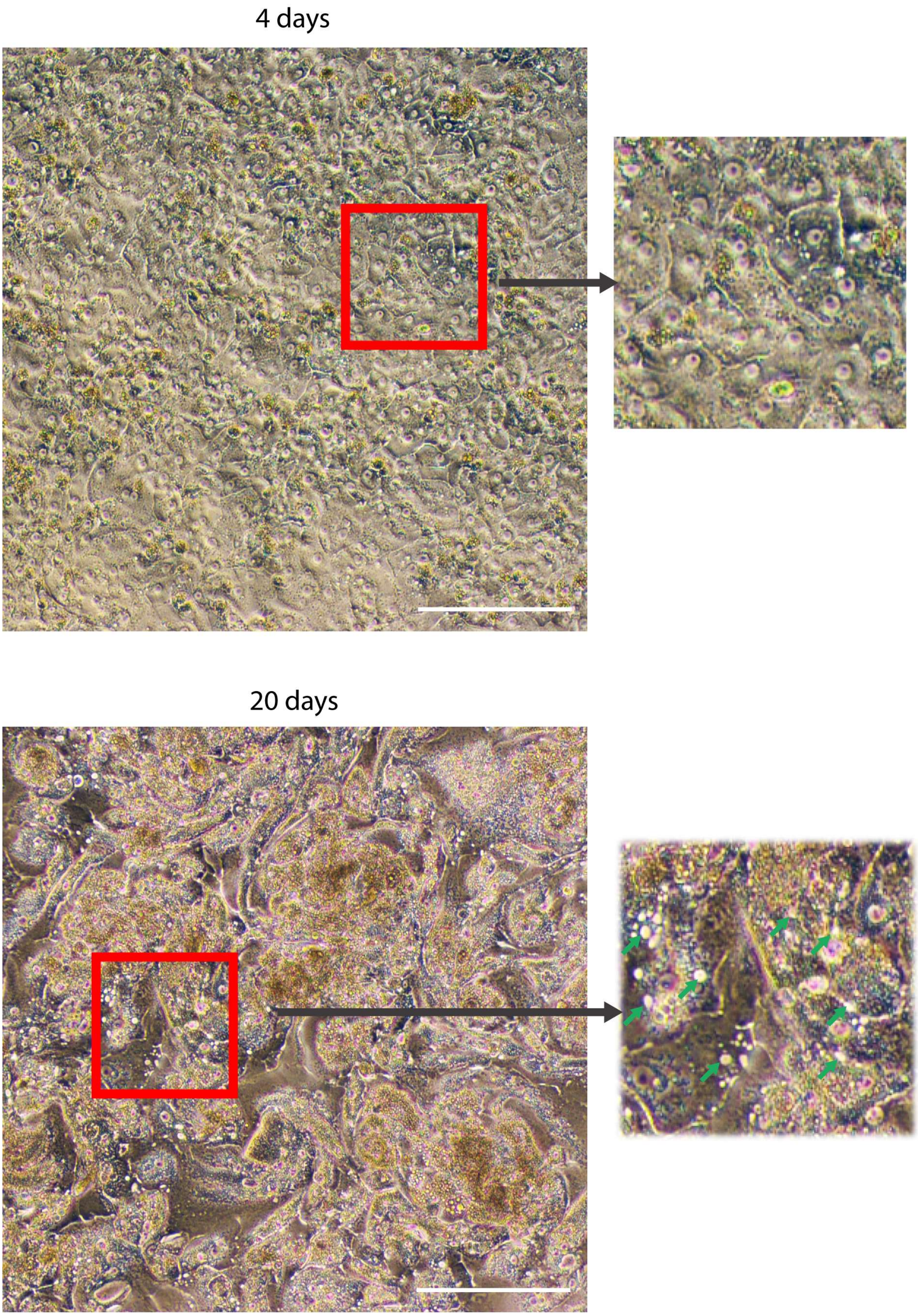

Figure S4. Primary human hepatocytes cultured in MG bilayer for 4 (upper panel) or 20 days (lower panel). Scale bars 100 µm. Green arrows (right panel) indicate vacuoles inside cells.

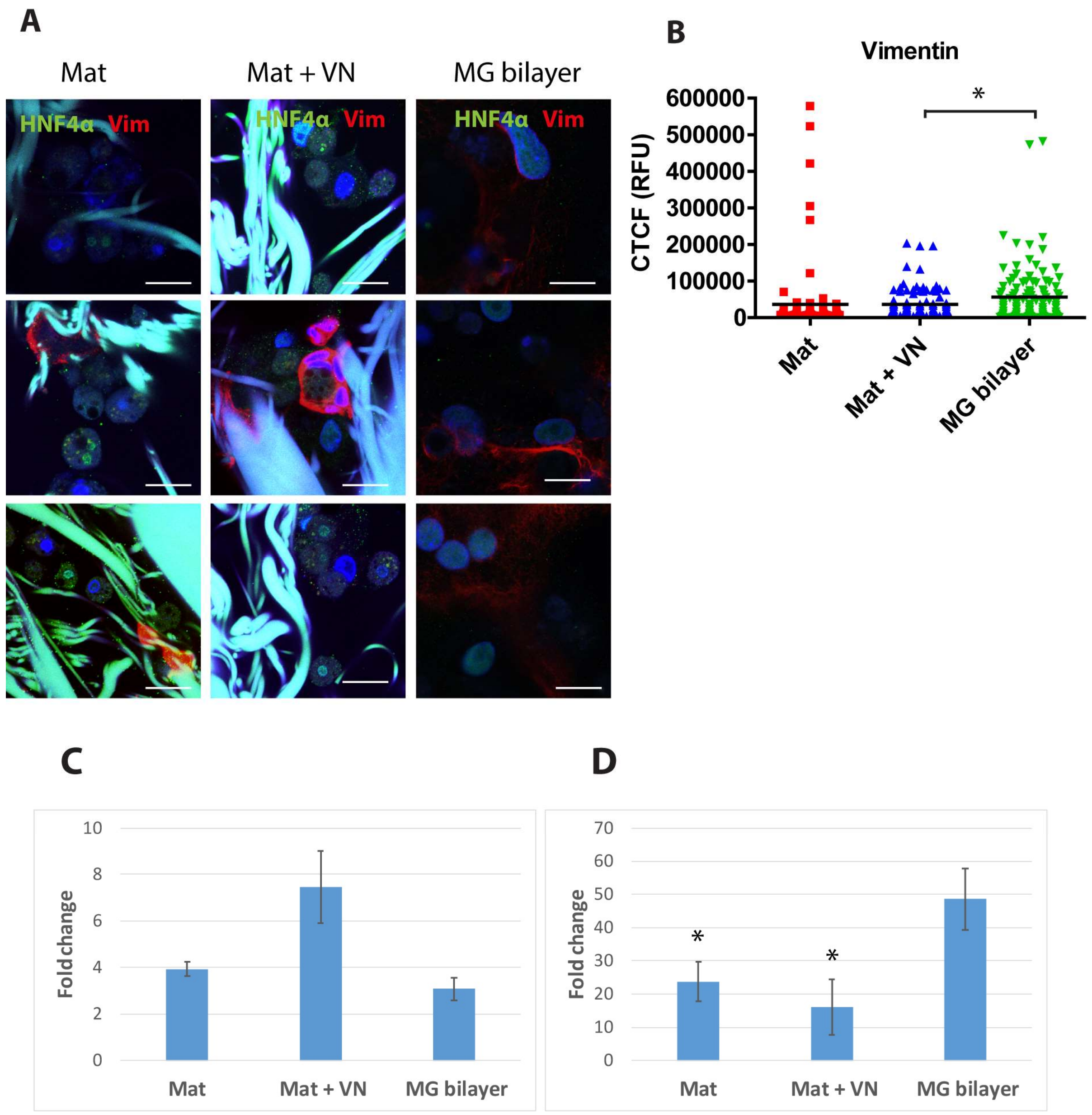

Figure S5. The differentiation status of primary human hepatocytes grown in long-term cultures. Cells were grown on SBS mats or as MG bilayer. (**A**) Expression of hepatocyte-specific transcription factor HNF4α (green) and mesenchymal (dedifferentiation) marker vimentin (red). Scale bars 20 µm. Three representative images from each sample (**A**) and quantification of vimentin expression (**B**). (**C**) RT-qPCR analysis of HNF4α expression; (**D**) RT-qPCR analysis of vimentin expression. * indicates a statistically significant (P<0.05) difference compared to MG bilayer.

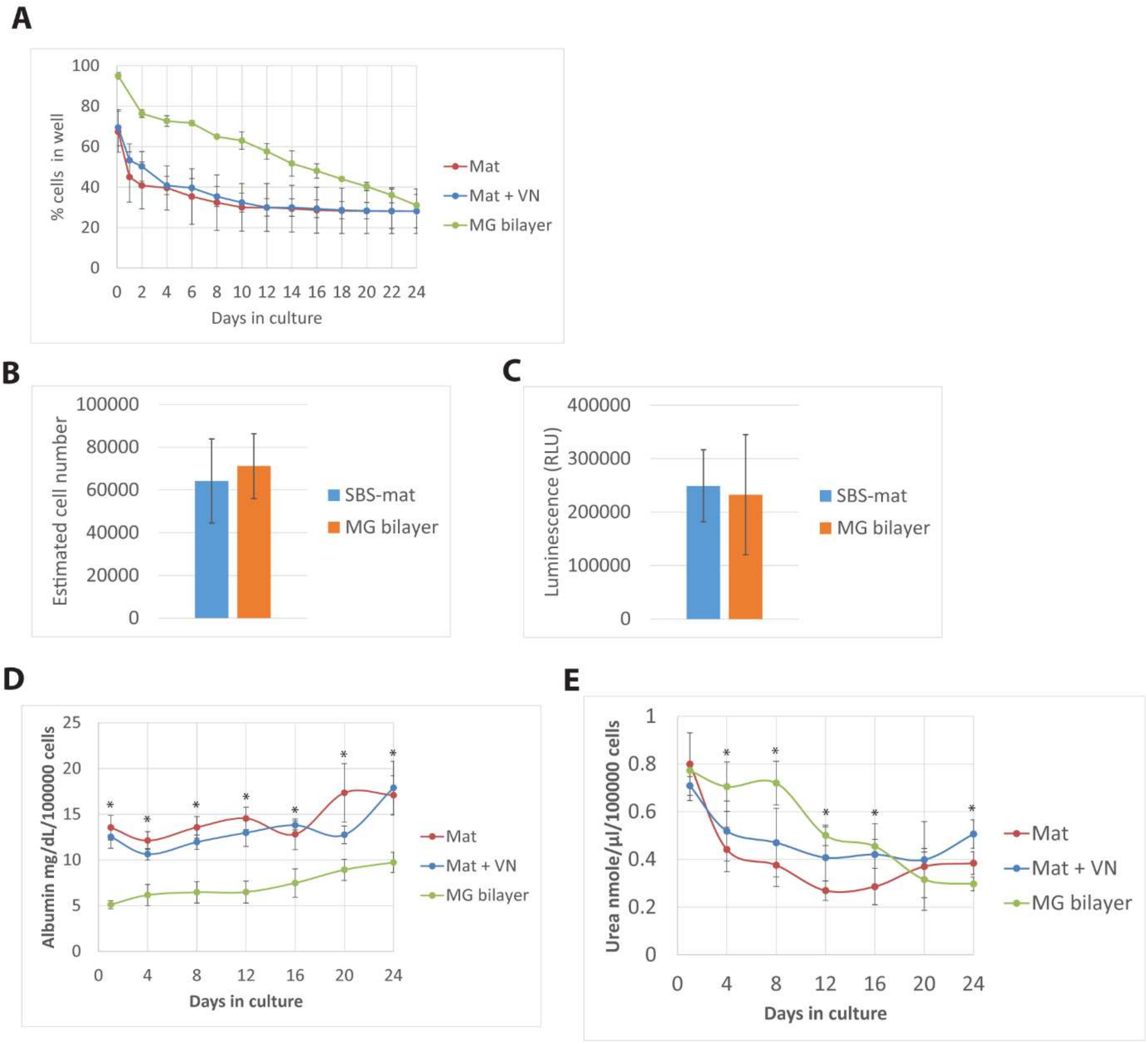

Figure S6. Estimated cell numbers and normalised metabolite production in long-term hepatocyte cultures. (**A**) Primary human hepatocyte attachment and viability in long-term cultures. The proportion of live attached cells in each well was estimated at 3 h after plating the cells and every 2 days thereafter, n=6. Estimated cell numbers at the final 24-day hepatocyte culture time-point (**B**) and validation of this cell number estimation by luminescence-conjugated viability assay CellTiter-Glo (**C**), n=6, RLU – relative light units. The estimated cell number normalised albumin (**D**) and urea (**E**) measurements in long-term hepatocyte cultures, n=5. * indicates a statistically significant ($P<0.05$) difference compared to MG bilayer.

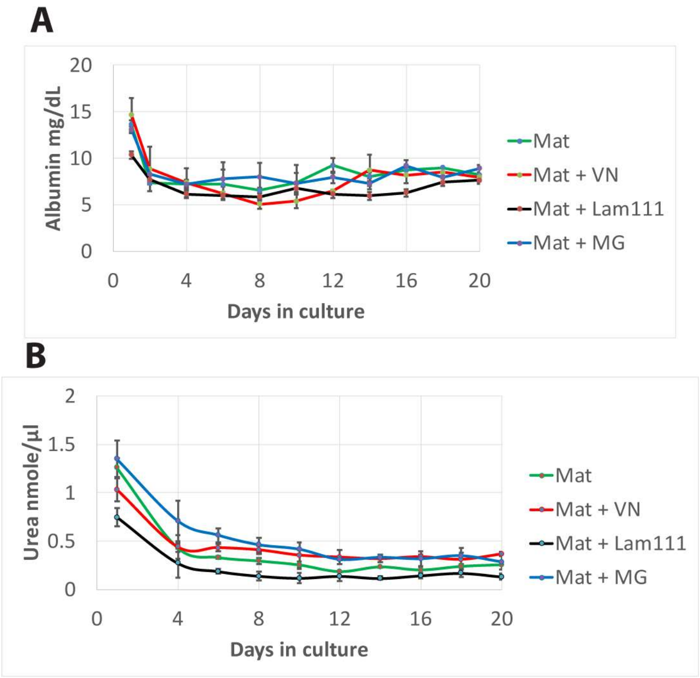

Figure S7. The dynamics of the synthesis of hepatocyte-specific metabolites albumin (**A**) and urea (**B**) was measured in the culture media of human primary hepatocytes. Data points show averages from 3 replicates +/- standard deviation.

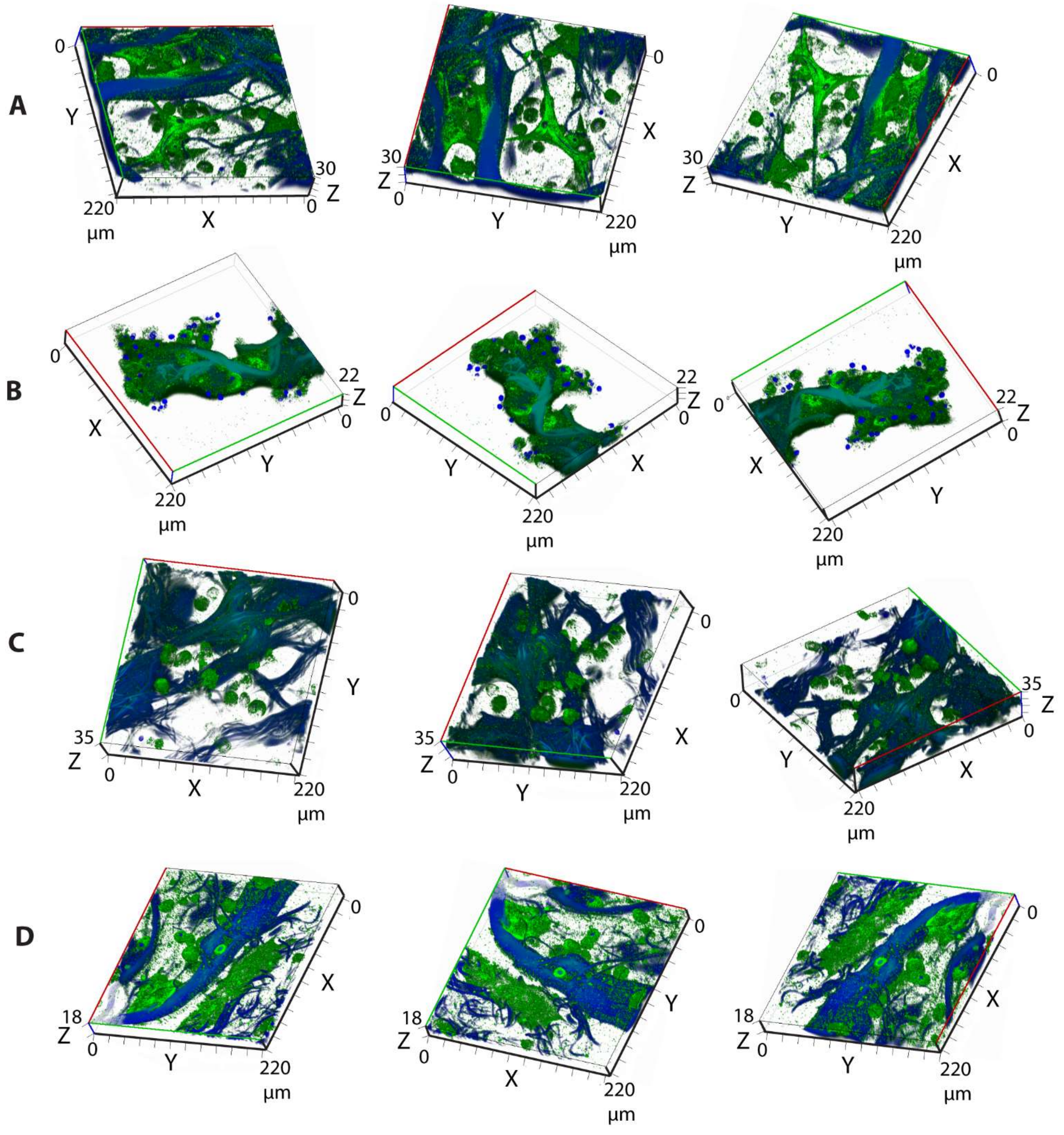

Figure S8. 3D images of the expression of ZO1 (green) in human primary hepatocytes grown on SBS mats depicted at 3 different projections. (**A**) SBS-mat and (**B**) vitronectin-coated mat at 4 days of culture; (**C**) SBS-mat and **(D)** vitronectin-coated mat at 20 days of culture. DAPI (blue) stains the cell nuclei and the mat fibers.

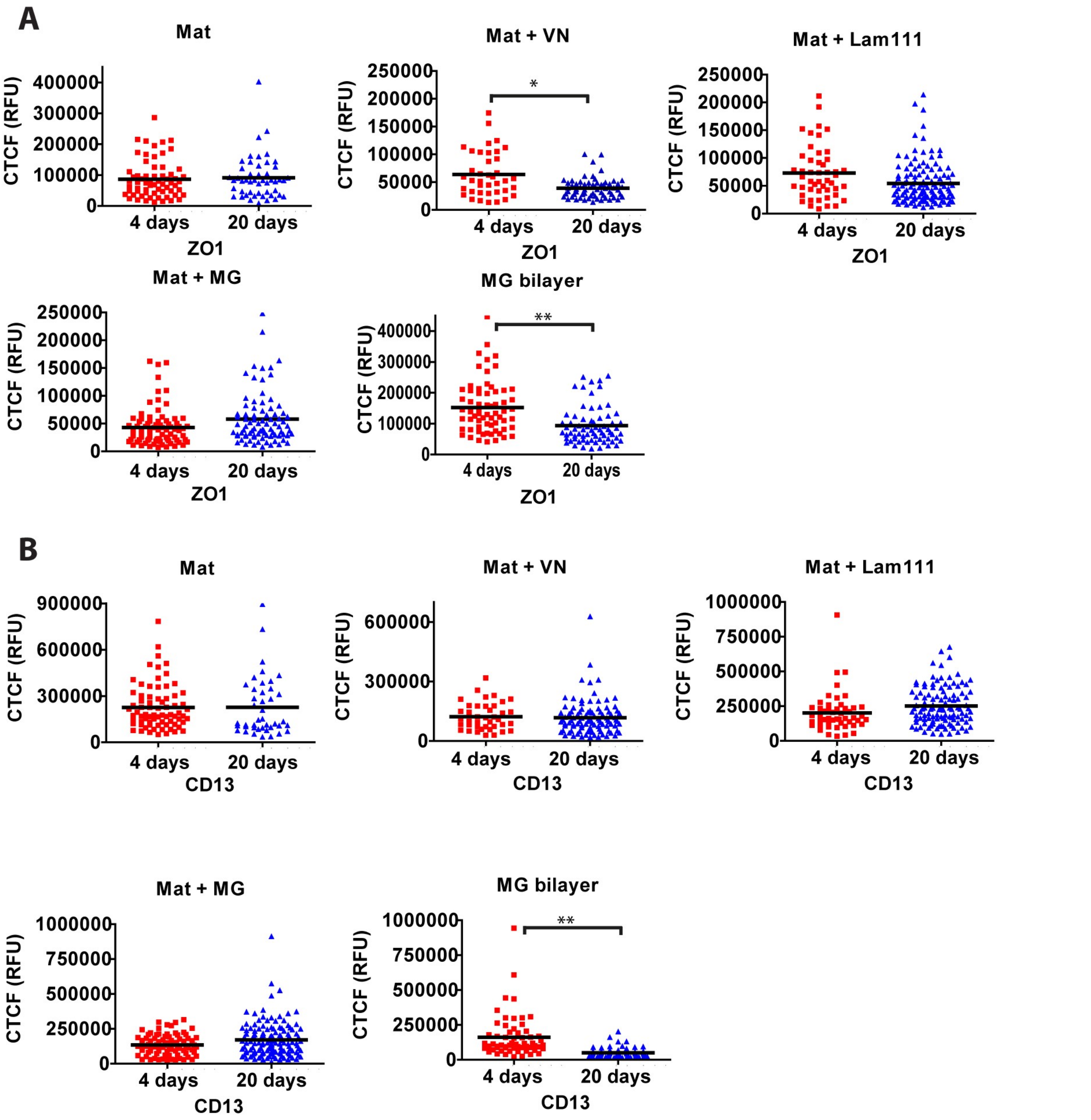

Figure S9. Quantification of the expression of cell polarization markers ZO1 (**A**) and CD13 (**B**) in human primary hepatocytes grown on SBS mats or MG bilayer. The level of CTCF (Corrected total cell fluorescence) was calculated based on confocal microscopy images. * indicates a statistically significant (P<0.05) difference, ** P< 0.001 compared to MG bilayer.

Reference

1 Klaas, M. *et al.* The alterations in the extracellular matrix composition guide the repair of damaged liver tissue. *Sci Rep* **6**, 27398, doi:10.1038/srep27398 (2016).
